# Diacetato{4′-[4-(benz­yloxy)phen­yl]-2,2′:6′,2′′-terpyridine}zinc(II)

**DOI:** 10.1107/S1600536809049502

**Published:** 2009-11-25

**Authors:** Wei Li, Zhan Guo Lu

**Affiliations:** a School of Food Engineering, Harbin University of Commerce, Harbin 150076, People’s Republic of China

## Abstract

In the title compound, [Zn(CH_3_COO)_2_(C_28_H_21_N_3_O)], the Zn^II^ ion is in a trigonal–bipyramidal ZnN_3_O_2_ coordination with a tridentate *N*-chelating 4′-[4-(benz­yloxy)phen­yl)-2,2′:6′,2′′-terpyridine ligand in the equatorial position and two acetate anions in the axial positions. The three pyridine rings are approximately coplanar, with a maximum deviation of 0.03 Å from the mean plane. The phen­oxy substituent makes a dihedral angle of 18.1 (2)° with the central pyridine ring. The benzyl group has a C—O—C—C torsion angle of 77.62 (8)° relative to the phen­oxy ring. In the crystal, mol­ecules are linked *via* C—H⋯O hydrogen bonds.

## Related literature

For the synthesis of functionalized terpyridines, see: Heller & Schubert (2003 ▶). For other structures with terpyridine ligands, see: Duprez *et al.* (2005 ▶). For a *trans*–*trans* arrangement of the pyridine rings about the inter­annular C—C bonds in the structure of a similar ligand, see: Anthonysamy *et al.* (2007 ▶). *PLATON* (Spek, 2009 ▶) was used for structure evaluation.
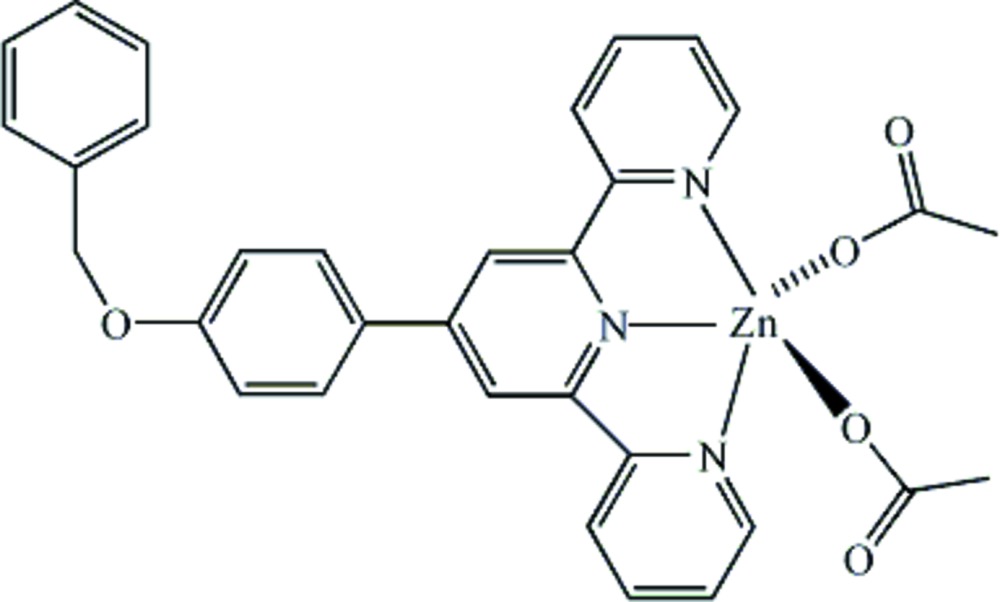


## Experimental

### 

#### Crystal data

[Zn(C_2_H_3_O_2_)_2_(C_28_H_21_N_3_O)]
*M* *_r_* = 598.94
Monoclinic, 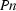

*a* = 8.3959 (17) Å
*b* = 15.564 (3) Å
*c* = 10.702 (2) Å
β = 102.23 (3)°
*V* = 1366.7 (5) Å^3^
*Z* = 2
Mo *K*α radiation
μ = 0.95 mm^−1^
*T* = 291 K
0.26 × 0.23 × 0.21 mm

#### Data collection

Rigaku R-AXIS RAPID diffractometer
Absorption correction: multi-scan (*ABSCOR*; Higashi, 1995 ▶) *T*_min_ = 0.791, *T*_max_ = 0.826
13194 measured reflections
5859 independent reflections
3538 reflections with *I* > 2σ(*I*)
*R*_int_ = 0.043

#### Refinement

*R*[*F* ^2^ > 2σ(*F* ^2^)] = 0.037
*wR*(*F* ^2^) = 0.106
*S* = 0.98
5859 reflections
373 parameters
2 restraints
H-atom parameters constrained
Δρ_max_ = 0.48 e Å^−3^
Δρ_min_ = −0.56 e Å^−3^
Absolute structure: Flack (1983 ▶), 2733 Friedel pairs
Flack parameter: 0.108 (13)

### 

Data collection: *RAPID-AUTO* (Rigaku, 1998 ▶); cell refinement: *RAPID-AUTO*; data reduction: *CrystalClear* (Rigaku/MSC, 2002 ▶); program(s) used to solve structure: *SHELXS97* (Sheldrick, 2008 ▶); program(s) used to refine structure: *SHELXL97* (Sheldrick, 2008 ▶); molecular graphics: *SHELXTL* (Sheldrick, 2008 ▶); software used to prepare material for publication: *SHELXL97*.

## Supplementary Material

Crystal structure: contains datablocks I, global. DOI: 10.1107/S1600536809049502/wm2284sup1.cif

Structure factors: contains datablocks I. DOI: 10.1107/S1600536809049502/wm2284Isup2.hkl

Additional supplementary materials:  crystallographic information; 3D view; checkCIF report

## Figures and Tables

**Table 1:** Selected bond lengths (Å)

Zn1—N1	2.187 (4)
Zn1—N2	2.090 (3)
Zn1—N3	2.151 (5)
Zn1—O2	2.014 (4)
Zn1—O4	1.961 (4)

**Table 2:** Hydrogen-bond geometry (Å, °)

*D*—H⋯*A*	*D*—H	H⋯*A*	*D*⋯*A*	*D*—H⋯*A*
C4—H4⋯O5^i^	0.93	2.39	3.293 (7)	163
C12—H12⋯O3^ii^	0.93	2.24	3.147 (7)	165
C22—H22*B*⋯O4^iii^	0.97	2.48	3.429 (6)	166

Symmetry codes: (i) 
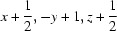
; (ii) 
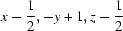
; (iii) 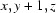.

**Fig. 1.**
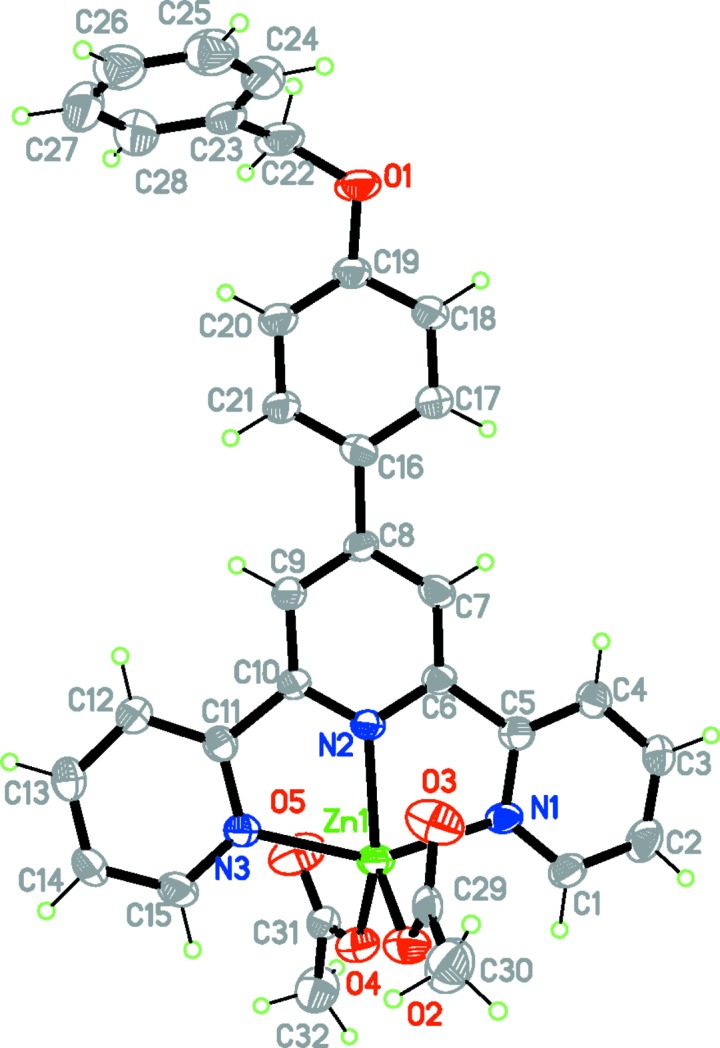
A view of the title compound (I) with the atomic numbering scheme. Displacement ellipsoids are drawn at the 50% probability level.

**Fig. 2.**
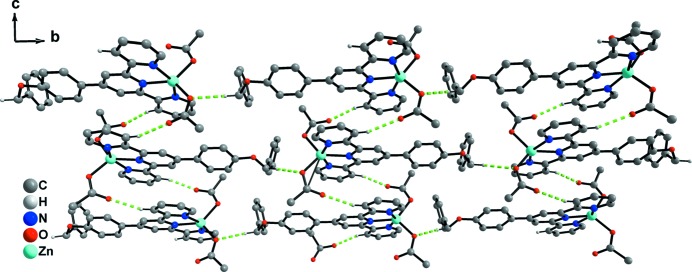
A view of a hydrogen-bonded (dashed lines) chain in (I). H atoms not involved in hydrogen bonding have been omitted.

## supplementary crystallographic information

### Comment

Functionalized terpyridines are interesting materials (Heller & Schubert, 2003). 2,2':6',2"-terpyridine and its derivatives have been used as key building blocks in supramolecular frameworks (Duprez *et al.*, 2005). We report here the crystal structure of the title compound, (I).

In the molecular structure of (I) (Fig. 1), the three pyridine rings are approximately coplanar (max. deviation from the mean plane 0.03 Å). The Zn^II^ ion is in a trigonal-bipyramidal coordination by a neutral tridentate 4'-[4-(benzyloxy)phenyl)-2,2':6',2"-terpyridine ligand in equatorial and two acetate anions in axial positions (Table 1). The phenoxy substituent makes a dihedral angle of 18.1 (2)° with the central pyridine ring. The torsion angle between the benzoyl group and the attached phenoxy ring, defined by atoms C19—O01—C22—C23, is 77.6 (7)°.

Molecules of (I) are linked *via* O—H···C hydrogen bonds, as shown in Fig. 2. Molecules associate *via* C(22)—H(22B)···O(4)#3 interactions, forming a chain along the *b* axis. Adjacent chains are interconnected *via* C(4)—H(4)···O(5)#1 and C(12)—H(12)···O(3)#2 interactions, leading to the formation of a two dimensional network (Table 2).

In the structure of a similar ligand molecule (Anthonysamy *et al.*, 2007) a *trans*-*trans* arrangement of the pyridine rings about the interannular C—C bonds is observed.

### Experimental

To a tetrahydrofuran solution (10 ml) of the ligand *L* (0.100 g, 0.241 mmol) was slowly added a methanolic solution (10 ml) of zinc acetate (0.044 g, 0.241 mmol). After stirring for 3 h at ambient temperature, a white solid was collected by filtration and washed with MeOH. Colorless single crystals suitable for X-ray determination were obtained by vapour diffusion of diethyl ether into a methanol solution of the powder sample over the course of 5 days.

### Refinement

Aromatic H atoms were fixed at C—H distances of 0.93 Å and refined as riding with *U*_iso_(H) = 1.2*U*_eq_(C). Other H atoms were positioned geometrically and refined using a riding model with C—H = 0.96–0.97 Å and with *U*_iso_(H) = 1.5 *U*_eq_(C). PLATON (Spek, 2009) suggests a (pseudo)-centrosymmetric structure in space group *P*2_1_/*c*. However, attempts to refine the structure in the centrosymmetric space group led to significantly higher residuals, high anisotropic displacement parameters and some disordered atoms. The crystal under investigation was refined as an inversion twin with a twin component ration of *ca.* 9:1.

### Crystal data

**Table:**

[Zn(C_2_H_3_O_2_)_2_(C_28_H_21_N_3_O)]	*F*(000) = 620
*M**_r_* = 598.94	*D*_x_ = 1.455 Mg m^−^^3^
Monoclinic, *P**n*	Mo *K*α radiation, λ = 0.71073 Å
Hall symbol: P -2yac	Cell parameters from 8985 reflections
*a* = 8.3959 (17) Å	θ = 3.1–27.4°
*b* = 15.564 (3) Å	µ = 0.95 mm^−^^1^
*c* = 10.702 (2) Å	*T* = 291 K
β = 102.23 (3)°	Block, colorless
*V* = 1366.7 (5) Å^3^	0.26 × 0.23 × 0.21 mm
*Z* = 2	

### Data collection

**Table:**

Rigaku R-AXIS RAPID diffractometer	5859 independent reflections
Radiation source: fine-focus sealed tube	3538 reflections with *I* > 2σ(*I*)
graphite	*R*_int_ = 0.043
ω scan	θ_max_ = 27.4°, θ_min_ = 3.1°
Absorption correction: multi-scan (*ABSCOR*; Higashi, 1995)	*h* = −10→10
*T*_min_ = 0.791, *T*_max_ = 0.826	*k* = −20→20
13194 measured reflections	*l* = −13→13

### Refinement

**Table:**

Refinement on *F*^2^	Secondary atom site location: difference Fourier map
Least-squares matrix: full	Hydrogen site location: inferred from neighbouring sites
*R*[*F*^2^ > 2σ(*F*^2^)] = 0.037	H-atom parameters constrained
*wR*(*F*^2^) = 0.106	*w* = 1/[σ^2^(*F*_o_^2^) + (0.0503*P*)^2^] where *P* = (*F*_o_^2^ + 2*F*_c_^2^)/3
*S* = 0.98	(Δ/σ)_max_ = 0.001
5859 reflections	Δρ_max_ = 0.48 e Å^−^^3^
373 parameters	Δρ_min_ = −0.56 e Å^−^^3^
2 restraints	Absolute structure: Flack (1983), 2733 Friedel pairs
Primary atom site location: structure-invariant direct methods	Flack parameter: 0.108 (13)

### Special details

**Table:**

Geometry. All e.s.d.'s (except the e.s.d. in the dihedral angle between two l.s. planes) are estimated using the full covariance matrix. The cell e.s.d.'s are taken into account individually in the estimation of e.s.d.'s in distances, angles and torsion angles; correlations between e.s.d.'s in cell parameters are only used when they are defined by crystal symmetry. An approximate (isotropic) treatment of cell e.s.d.'s is used for estimating e.s.d.'s involving l.s. planes.
Refinement. Refinement of *F*^2^ against ALL reflections. The weighted *R*-factor *wR* and goodness of fit *S* are based on *F*^2^, conventional *R*-factors *R* are based on *F*, with *F* set to zero for negative *F*^2^. The threshold expression of *F*^2^ > σ(*F*^2^) is used only for calculating *R*-factors(gt) *etc*. and is not relevant to the choice of reflections for refinement. *R*-factors based on *F*^2^ are statistically about twice as large as those based on *F*, and *R*- factors based on ALL data will be even larger.

### Fractional atomic coordinates and isotropic or equivalent isotropic displacement parameters (Å^2^)

**Table:**

	*x*	*y*	*z*	*U*_iso_*/*U*_eq_	
C1	0.8831 (7)	0.3046 (4)	0.9729 (6)	0.0593 (17)	
H1	0.8640	0.2457	0.9695	0.071*	
C2	1.0368 (7)	0.3342 (4)	1.0203 (7)	0.071 (2)	
H2	1.1225	0.2956	1.0432	0.085*	
C3	1.0655 (6)	0.4200 (4)	1.0342 (5)	0.0584 (15)	
H3	1.1690	0.4404	1.0707	0.070*	
C4	0.9378 (6)	0.4766 (4)	0.9929 (5)	0.0547 (14)	
H4	0.9538	0.5357	0.9989	0.066*	
C5	0.7855 (6)	0.4424 (3)	0.9424 (5)	0.0457 (12)	
C6	0.6379 (6)	0.4966 (3)	0.8920 (5)	0.0436 (13)	
C7	0.6359 (6)	0.5845 (3)	0.8898 (5)	0.0505 (14)	
H7	0.7320	0.6150	0.9187	0.061*	
C8	0.4896 (7)	0.6291 (2)	0.8440 (7)	0.0441 (10)	
C9	0.3513 (6)	0.5806 (3)	0.8006 (5)	0.0478 (13)	
H9	0.2518	0.6076	0.7702	0.057*	
C10	0.3608 (6)	0.4912 (3)	0.8023 (5)	0.0423 (13)	
C11	0.2215 (6)	0.4320 (3)	0.7578 (5)	0.0475 (13)	
C12	0.0654 (6)	0.4599 (4)	0.7014 (5)	0.0571 (15)	
H12	0.0426	0.5181	0.6880	0.068*	
C13	−0.0562 (7)	0.3980 (4)	0.6655 (6)	0.0645 (17)	
H13	−0.1622	0.4148	0.6292	0.077*	
C14	−0.0200 (7)	0.3134 (4)	0.6834 (6)	0.0600 (16)	
H14	−0.0997	0.2718	0.6585	0.072*	
C15	0.1373 (7)	0.2905 (4)	0.7393 (6)	0.0615 (16)	
H15	0.1606	0.2325	0.7536	0.074*	
C16	0.4862 (7)	0.7251 (2)	0.8434 (6)	0.0482 (11)	
C17	0.6114 (7)	0.7729 (4)	0.9184 (6)	0.0603 (15)	
H17	0.6995	0.7449	0.9696	0.072*	
C18	0.6047 (7)	0.8626 (4)	0.9167 (6)	0.0638 (16)	
H18	0.6878	0.8943	0.9676	0.077*	
C19	0.4755 (6)	0.9042 (3)	0.8400 (6)	0.0562 (14)	
C20	0.3523 (6)	0.8577 (3)	0.7660 (6)	0.0611 (15)	
H20	0.2646	0.8859	0.7146	0.073*	
C21	0.3582 (6)	0.7699 (3)	0.7674 (5)	0.0573 (14)	
H21	0.2740	0.7392	0.7161	0.069*	
C22	0.3569 (6)	1.0418 (3)	0.7676 (6)	0.0794 (14)	
H22A	0.3336	1.0169	0.6825	0.095*	
H22B	0.3954	1.1000	0.7606	0.095*	
C23	0.2019 (6)	1.0450 (3)	0.8165 (5)	0.0635 (13)	
C24	0.2036 (7)	1.0670 (3)	0.9414 (5)	0.0758 (14)	
H24	0.3027	1.0775	0.9972	0.091*	
C25	0.0619 (8)	1.0737 (4)	0.9849 (7)	0.0897 (18)	
H25	0.0673	1.0886	1.0699	0.108*	
C26	−0.0850 (8)	1.0595 (4)	0.9082 (8)	0.0888 (19)	
H26	−0.1798	1.0633	0.9397	0.107*	
C27	−0.0913 (7)	1.0392 (4)	0.7829 (8)	0.100 (2)	
H27	−0.1920	1.0308	0.7283	0.120*	
C28	0.0516 (8)	1.0309 (4)	0.7351 (6)	0.0859 (18)	
H28	0.0456	1.0161	0.6500	0.103*	
C29	0.4567 (6)	0.2751 (4)	1.0846 (6)	0.0572 (15)	
C30	0.4161 (8)	0.2233 (5)	1.1989 (7)	0.100 (2)	
H30A	0.3133	0.1947	1.1716	0.149*	
H30B	0.4099	0.2619	1.2676	0.149*	
H30C	0.4999	0.1815	1.2276	0.149*	
C31	0.5525 (6)	0.2511 (4)	0.6214 (6)	0.0605 (15)	
C32	0.5948 (9)	0.1794 (4)	0.5365 (6)	0.086 (2)	
H32A	0.4985	0.1626	0.4757	0.129*	
H32B	0.6371	0.1309	0.5885	0.129*	
H32C	0.6754	0.1997	0.4919	0.129*	
N1	0.7606 (5)	0.3578 (3)	0.9319 (5)	0.0481 (11)	
N2	0.5019 (6)	0.45091 (16)	0.8450 (5)	0.0440 (7)	
N3	0.2591 (6)	0.3473 (3)	0.7745 (5)	0.0523 (12)	
O1	0.4850 (6)	0.99246 (16)	0.8480 (6)	0.0784 (10)	
O2	0.4764 (5)	0.2367 (3)	0.9965 (4)	0.0664 (11)	
O3	0.4605 (6)	0.3537 (3)	1.1031 (5)	0.0977 (14)	
O4	0.5539 (5)	0.2307 (2)	0.7346 (4)	0.0696 (11)	
O5	0.5191 (7)	0.3222 (3)	0.5782 (5)	0.1088 (18)	
Zn1	0.50944 (8)	0.31689 (2)	0.85645 (8)	0.05098 (14)	

### Atomic displacement parameters (Å^2^)

**Table:**

	*U*^11^	*U*^22^	*U*^33^	*U*^12^	*U*^13^	*U*^23^
C1	0.049 (3)	0.046 (3)	0.082 (4)	0.007 (2)	0.012 (3)	0.009 (3)
C2	0.042 (3)	0.078 (4)	0.092 (5)	0.017 (3)	0.010 (3)	0.010 (3)
C3	0.042 (3)	0.066 (3)	0.063 (3)	−0.001 (3)	0.002 (2)	0.000 (3)
C4	0.043 (3)	0.056 (3)	0.065 (3)	0.001 (2)	0.011 (2)	−0.004 (3)
C5	0.040 (3)	0.044 (3)	0.056 (3)	−0.001 (2)	0.017 (2)	0.000 (2)
C6	0.039 (3)	0.037 (3)	0.056 (3)	0.003 (2)	0.013 (2)	−0.005 (2)
C7	0.046 (3)	0.042 (3)	0.064 (3)	−0.009 (2)	0.013 (2)	−0.003 (2)
C8	0.043 (3)	0.0359 (16)	0.055 (3)	0.003 (2)	0.013 (2)	0.001 (3)
C9	0.037 (3)	0.041 (3)	0.065 (3)	0.000 (2)	0.010 (2)	0.000 (2)
C10	0.040 (3)	0.037 (3)	0.049 (3)	0.004 (2)	0.009 (2)	−0.003 (2)
C11	0.040 (3)	0.047 (3)	0.057 (3)	−0.004 (2)	0.012 (2)	0.003 (2)
C12	0.050 (3)	0.051 (3)	0.068 (4)	0.004 (2)	0.007 (3)	0.003 (3)
C13	0.039 (3)	0.071 (4)	0.081 (4)	−0.005 (3)	0.007 (2)	−0.001 (3)
C14	0.048 (3)	0.055 (3)	0.073 (4)	−0.014 (2)	0.005 (3)	−0.009 (2)
C15	0.053 (3)	0.045 (3)	0.088 (5)	−0.011 (3)	0.017 (3)	0.001 (3)
C16	0.051 (3)	0.0395 (18)	0.057 (3)	−0.007 (2)	0.018 (2)	−0.007 (3)
C17	0.053 (3)	0.044 (3)	0.080 (4)	0.002 (2)	0.005 (3)	−0.002 (3)
C18	0.050 (3)	0.043 (3)	0.093 (4)	−0.004 (2)	0.004 (3)	−0.010 (3)
C19	0.050 (4)	0.0365 (19)	0.082 (4)	−0.002 (2)	0.014 (3)	0.000 (3)
C20	0.052 (3)	0.039 (3)	0.092 (4)	0.001 (2)	0.014 (3)	0.005 (3)
C21	0.053 (3)	0.039 (3)	0.077 (4)	−0.001 (2)	0.008 (2)	0.007 (3)
C22	0.082 (4)	0.039 (2)	0.122 (4)	0.002 (2)	0.031 (3)	0.009 (3)
C23	0.061 (3)	0.034 (2)	0.093 (4)	0.007 (2)	0.011 (3)	0.007 (2)
C24	0.065 (3)	0.065 (3)	0.095 (4)	−0.001 (3)	0.013 (3)	0.000 (3)
C25	0.088 (5)	0.080 (4)	0.109 (5)	0.003 (3)	0.038 (4)	−0.008 (3)
C26	0.081 (5)	0.067 (4)	0.124 (6)	−0.004 (3)	0.036 (4)	−0.001 (4)
C27	0.059 (4)	0.087 (5)	0.144 (7)	0.006 (3)	0.001 (4)	0.009 (4)
C28	0.082 (4)	0.080 (4)	0.090 (5)	0.000 (3)	0.008 (3)	0.001 (3)
C29	0.031 (2)	0.059 (4)	0.074 (4)	−0.007 (2)	−0.006 (2)	0.012 (3)
C30	0.076 (4)	0.106 (6)	0.113 (5)	−0.001 (4)	0.013 (4)	0.036 (5)
C31	0.045 (3)	0.048 (3)	0.084 (4)	0.003 (2)	0.002 (2)	−0.002 (3)
C32	0.089 (4)	0.094 (5)	0.074 (4)	0.022 (4)	0.017 (3)	−0.017 (3)
N1	0.040 (2)	0.037 (2)	0.071 (3)	0.0050 (19)	0.020 (2)	0.000 (2)
N2	0.0421 (15)	0.0334 (13)	0.056 (2)	−0.001 (2)	0.0103 (14)	−0.002 (2)
N3	0.047 (3)	0.040 (2)	0.068 (3)	0.000 (2)	0.010 (2)	−0.002 (2)
O1	0.062 (3)	0.0335 (13)	0.141 (3)	−0.0014 (18)	0.024 (2)	0.000 (3)
O2	0.072 (3)	0.049 (2)	0.083 (3)	−0.0032 (19)	0.027 (2)	0.004 (2)
O3	0.113 (3)	0.073 (3)	0.112 (3)	−0.017 (3)	0.034 (3)	−0.019 (3)
O4	0.078 (3)	0.049 (2)	0.080 (3)	0.008 (2)	0.014 (2)	−0.001 (2)
O5	0.138 (5)	0.077 (3)	0.118 (4)	0.024 (3)	0.042 (3)	0.026 (3)
Zn1	0.0465 (2)	0.0366 (2)	0.0702 (3)	0.0007 (4)	0.01314 (18)	0.0003 (4)

### Geometric parameters (Å, °)

**Table:**

C1—N1	1.320 (7)	C19—C20	1.369 (7)
C1—C2	1.363 (9)	C19—O1	1.377 (5)
C1—H1	0.9300	C20—C21	1.369 (8)
C2—C3	1.359 (8)	C20—H20	0.9300
C2—H2	0.9300	C21—H21	0.9300
C3—C4	1.385 (7)	C22—O1	1.448 (6)
C3—H3	0.9300	C22—C23	1.503 (7)
C4—C5	1.385 (7)	C22—H22A	0.9700
C4—H4	0.9300	C22—H22B	0.9700
C5—N1	1.334 (7)	C23—C24	1.377 (7)
C5—C6	1.503 (7)	C23—C28	1.391 (7)
C6—N2	1.349 (6)	C24—C25	1.370 (8)
C6—C7	1.368 (8)	C24—H24	0.9300
C7—C8	1.406 (7)	C25—C26	1.347 (8)
C7—H7	0.9300	C25—H25	0.9300
C8—C9	1.381 (7)	C26—C27	1.367 (9)
C8—C16	1.494 (5)	C26—H26	0.9300
C9—C10	1.394 (8)	C27—C28	1.406 (9)
C9—H9	0.9300	C27—H27	0.9300
C10—N2	1.333 (6)	C28—H28	0.9300
C10—C11	1.486 (7)	C29—O2	1.158 (6)
C11—N3	1.359 (7)	C29—O3	1.238 (7)
C11—C12	1.391 (7)	C29—C30	1.562 (8)
C12—C13	1.397 (7)	C30—H30A	0.9600
C12—H12	0.9300	C30—H30B	0.9600
C13—C14	1.355 (8)	C30—H30C	0.9600
C13—H13	0.9300	C31—O5	1.209 (7)
C14—C15	1.376 (8)	C31—O4	1.249 (7)
C14—H14	0.9300	C31—C32	1.527 (8)
C15—N3	1.344 (7)	C32—H32A	0.9600
C15—H15	0.9300	C32—H32B	0.9600
C16—C21	1.390 (7)	C32—H32C	0.9600
C16—C17	1.394 (7)	Zn1—N1	2.187 (4)
C17—C18	1.399 (9)	Zn1—N2	2.090 (3)
C17—H17	0.9300	Zn1—N3	2.151 (5)
C18—C19	1.376 (7)	Zn1—O2	2.014 (4)
C18—H18	0.9300	Zn1—O4	1.961 (4)
			
N1—C1—C2	121.4 (6)	O1—C22—H22A	108.9
N1—C1—H1	119.3	C23—C22—H22A	108.9
C2—C1—H1	119.3	O1—C22—H22B	108.9
C3—C2—C1	120.3 (6)	C23—C22—H22B	108.9
C3—C2—H2	119.9	H22A—C22—H22B	107.7
C1—C2—H2	119.9	C24—C23—C28	118.0 (5)
C2—C3—C4	118.8 (5)	C24—C23—C22	121.0 (5)
C2—C3—H3	120.6	C28—C23—C22	120.9 (5)
C4—C3—H3	120.6	C25—C24—C23	121.1 (5)
C5—C4—C3	117.9 (6)	C25—C24—H24	119.4
C5—C4—H4	121.0	C23—C24—H24	119.4
C3—C4—H4	121.0	C26—C25—C24	122.0 (7)
N1—C5—C4	121.8 (5)	C26—C25—H25	119.0
N1—C5—C6	115.0 (4)	C24—C25—H25	119.0
C4—C5—C6	123.2 (5)	C25—C26—C27	118.4 (6)
N2—C6—C7	121.0 (5)	C25—C26—H26	120.8
N2—C6—C5	113.9 (4)	C27—C26—H26	120.8
C7—C6—C5	125.1 (5)	C26—C27—C28	121.3 (5)
C6—C7—C8	120.4 (5)	C26—C27—H27	119.3
C6—C7—H7	119.8	C28—C27—H27	119.3
C8—C7—H7	119.8	C23—C28—C27	119.2 (6)
C9—C8—C7	117.3 (3)	C23—C28—H28	120.4
C9—C8—C16	122.1 (5)	C27—C28—H28	120.4
C7—C8—C16	120.6 (5)	O2—C29—O3	129.6 (6)
C8—C9—C10	120.0 (5)	O2—C29—C30	117.6 (5)
C8—C9—H9	120.0	O3—C29—C30	112.8 (6)
C10—C9—H9	120.0	C29—C30—H30A	109.5
N2—C10—C9	121.2 (5)	C29—C30—H30B	109.5
N2—C10—C11	113.6 (5)	H30A—C30—H30B	109.5
C9—C10—C11	125.2 (5)	C29—C30—H30C	109.5
N3—C11—C12	122.1 (5)	H30A—C30—H30C	109.5
N3—C11—C10	114.4 (4)	H30B—C30—H30C	109.5
C12—C11—C10	123.4 (5)	O5—C31—O4	123.8 (6)
C11—C12—C13	118.1 (6)	O5—C31—C32	120.4 (7)
C11—C12—H12	121.0	O4—C31—C32	115.8 (5)
C13—C12—H12	121.0	C31—C32—H32A	109.5
C14—C13—C12	120.1 (5)	C31—C32—H32B	109.5
C14—C13—H13	119.9	H32A—C32—H32B	109.5
C12—C13—H13	119.9	C31—C32—H32C	109.5
C13—C14—C15	118.6 (6)	H32A—C32—H32C	109.5
C13—C14—H14	120.7	H32B—C32—H32C	109.5
C15—C14—H14	120.7	C1—N1—C5	119.6 (5)
N3—C15—C14	123.7 (6)	C1—N1—Zn1	124.2 (4)
N3—C15—H15	118.1	C5—N1—Zn1	116.1 (3)
C14—C15—H15	118.1	C10—N2—C6	120.0 (3)
C21—C16—C17	117.7 (4)	C10—N2—Zn1	120.2 (4)
C21—C16—C8	121.0 (5)	C6—N2—Zn1	119.5 (4)
C17—C16—C8	121.3 (5)	C15—N3—C11	117.3 (5)
C16—C17—C18	120.2 (5)	C15—N3—Zn1	126.1 (4)
C16—C17—H17	119.9	C11—N3—Zn1	116.6 (3)
C18—C17—H17	119.9	C19—O1—C22	117.8 (4)
C19—C18—C17	120.1 (5)	C29—O2—Zn1	110.5 (4)
C19—C18—H18	119.9	C31—O4—Zn1	120.5 (4)
C17—C18—H18	119.9	O4—Zn1—O2	98.42 (13)
C20—C19—C18	120.0 (4)	O4—Zn1—N2	130.6 (2)
C20—C19—O1	126.1 (5)	O2—Zn1—N2	130.9 (2)
C18—C19—O1	113.8 (5)	O4—Zn1—N3	100.76 (19)
C21—C20—C19	120.0 (5)	O2—Zn1—N3	99.39 (18)
C21—C20—H20	120.0	N2—Zn1—N3	75.02 (19)
C19—C20—H20	120.0	N4—Zn1—N1	98.04 (17)
C20—C21—C16	122.0 (5)	O2—Zn1—N1	100.36 (18)
C20—C21—H21	119.0	N2—Zn1—N1	75.28 (18)
C16—C21—H21	119.0	N3—Zn1—N1	150.30 (11)
O1—C22—C23	113.5 (5)		

### Hydrogen-bond geometry (Å, °)

**Table:**

*D*—H···*A*	*D*—H	H···*A*	*D*···*A*	*D*—H···*A*
C4—H4···O5^i^	0.93	2.39	3.293 (7)	163
C12—H12···O3^ii^	0.93	2.24	3.147 (7)	165
C22—H22B···O4^iii^	0.97	2.48	3.429 (6)	166

Symmetry codes: (i) *x*+1/2, −*y*+1, *z*+1/2; (ii) *x*−1/2, −*y*+1, *z*−1/2; (iii) *x*, *y*+1, *z*.

## References

[bb1] Anthonysamy, A., Balasubramanian, S., Chinnakali, K. & Fun, H.-K. (2007). *Acta Cryst.* E**63**, o1148–o1150.

[bb2] Duprez, V., Biancardo, M., Spanggaard, H. & Krebs, F. C. (2005). *Macromolecules*, **38**, 10436–10448.

[bb3] Flack, H. D. (1983). *Acta Cryst.* A**39**, 876–881.

[bb4] Heller, M. & Schubert, U. S. (2003). *Eur. J. Org. Chem.* pp. 947–961.

[bb5] Higashi, T. (1995). *ABSCOR*. Rigaku Corporation, Tokyo, Japan.

[bb6] Rigaku (1998). *RAPID-AUTO*. Rigaku Corporation, Tokyo, Japan.

[bb7] Rigaku/MSC (2002). *CrystalStructure.* Rigaku/MSC Inc., The Woodlands, Texas, USA.

[bb8] Sheldrick, G. M. (2008). *Acta Cryst.* A**64**, 112–122. 10.1107/S0108767307043930 18156677

[bb9] Spek, A. L. (2009). *Acta Cryst.* D**65**, 148–155. 10.1107/S090744490804362X PMC2631630 19171970

